# Complete genome sequence of sixteen plant growth promoting *Streptomyces* strains

**DOI:** 10.1038/s41598-020-67153-9

**Published:** 2020-06-24

**Authors:** Gopalakrishnan Subramaniam, Vivek Thakur, Rachit K. Saxena, Srinivas Vadlamudi, Shilp Purohit, Vinay Kumar, Abhishek Rathore, Annapurna Chitikineni, Rajeev K. Varshney

**Affiliations:** 1 International Crops Research Institute for the Semi-Arid Tropics, Hyderabad, India; 2 School of Life Sciences, University of Hyderabad, Hyderabad, India

**Keywords:** Applied microbiology, Bacterial genes

## Abstract

The genome sequences of 16 *Streptomyces* strains, showing potential for plant growth-promotion (PGP) activities in rice, sorghum, chickpea and pigeonpea, isolated from herbal vermicompost, have been decoded. The genome assemblies of the 16 *Streptomyces* strains ranged from 6.8 Mb to 8.31 Mb, with a GC content of 72 to 73%. The extent of sequence similarity (in terms of shared ortholog) in 16 *Streptomyces* strains showed 70 to 85% common genes to the closest publicly available *Streptomyces* genomes. It was possible to identify ~1,850 molecular functions across these 16 strains, of which close to 50% were conserved across the genomes of *Streptomyces* strains, whereas, ~10% were strain specific and the rest were present in various combinations. Genome assemblies of the 16 *Streptomyces* strains have also provided genes involved in key pathways related to PGP and biocontrol traits such as siderophores, auxin, hydrocyanic acid, chitinase and cellulase. Further, the genome assemblies provided better understanding of genetic similarity among target strains and with the publically available *Streptomyces* strains.

## Introduction

Plants attract beneficial microbes through their root exudates and such associative or symbiotic microbiomes in-turn induces the plant fitness and immune system via cell-signaling and/or by triggering plant’s physiological process. Microbiome in vicinity of roots plays an important role in plant growth, development and abiotic/biotic stress tolerance^1,2^. Unraveling of plant-microbiome interaction develops a basis for sustainable strategies in next-generation farming with less input of fertilizers and/or pesticides^3^. Among the plant-associated microbes, actinobacteria are of particular interest due to their ability to produce a range of secondary metabolites^4^. Actinobacteria have been found to be associated with biological control of insect pests and pathogens, stress tolerance and growth promotion in plants^1,2^. They occur in the rhizosphere as well as with in the plants (in the form of endophytes) and have been shown to induce systemic resistance in plants. Among the actinobacteria, *Streptomyces* is the predominant genus followed by *Actinomadura*, *Microbispora*, *Micromonospora*, *Nocardia*, *Nonomurea*, *Mycobacterium, Frankia, Actinoplanes, Saccharopolyspora* and *Verrucosispora* and are known for their ubiquitous presence in soil and nutrient cycling; and majorly for the antibiotics and complex secondary metabolite pathways^5,6^. *Streptomyces* is the major producer of secondary metabolites (39% of the total metabolites produced by the microbes) including polyene macrolides, actinomycins, aminoglycosides, streptothricins, anthracyclines, cyclopolylactones and quinoxaline-peptides^5^. The chemical diversity of metabolites produced by *Streptomyces* ranges from simple lactones to condensed macro-lactones; and simple amino acid derivatives to peptides and high-molecular-weight proteins. A broad range of *Streptomyces* activities on pharmacological traits have been characterized but the traits related to agriculture have received relatively less consideration. Hence, exploring *Streptomyces* for agricultural sector becomes an area of active interest in current scenario^7,8^.

Previously, we had identified 16 candidate *Streptomyces* strains (through 16 S rDNA sequencing method) and characterized for their plant growth promotion (PGP) traits such as indole acetic acid, siderophore, β-1,3-glucanase, chitinase, hydrocyanic acid and other hydrolytic enzymes^9–23^ (Table 1). Characterizations of these microbes have provided information on their beneficial traits and were further demonstrated for PGP activities and antagonistic activities (against pathogens of chickpea, pigeonpea and sorghum) *in planta*^9–23^. Some of these *Streptomyces* strains were also demonstrated for their larvicidal activity against *Helicoverpa armigera*, and other important caterpillar pests such as *Spodoptera litura* and *Chilo partellus*^15^. From these collections, two metabolites with insecticidal activity against *H. armigera* such as, a diketopiperazine derivative called cyclo (Trp‐Phe), and a novel fatty acid amide derivative called N‐(1‐(2,2‐dimethyl‐5‐undecyl‐1,3‐dioxolan‐4‐yl)‐2‐hydroxyethyl) stearamide were isolated and characterized from the best strains *S. griseoplanus* SAI‐25 and *Streptomyces* sp., CAI‐155, respectively^21,22^. However, the detailed molecular characterization of the above mentioned *Streptomyces* strains have not been done, so far.

**Table 1:** Details of 16 *Streptomyces* strains and their PGP traits.

S.no	Strain Number	Identification by 16S rDNA sequencing	Gene bank ACC. No.	*In vitro* PGP traits						Antifungal activity					Crops	Reference
				Indole Acetic acid (μg ml^−1^)	Sidero phore”	Hydrocy anic acid#	Cellu lase!	Chiti nase!	β 1,3 glucanase (units)	FOC	Mp	Rb	Bc	Sr		
1	CAI-17	*Streptomyces* sp.	JQ682619	0.34	2	3	+	+	0.66	+	+	+	+	−	R,S,C	10,12,17,21
2	CAI-21	*Streptomyces* sp.	JQ682620	1.13	1	3	+	−	0	+	+	−	−	+	R,S,C,P	10,11,18,21,23
3	CAI-24	*Streptomyces* sp.	JN400112	5.90	3	3	+	+	0	+	−	+	+	−	R,S,C	9,14,16,19,21
4	CAI-68	*Streptomyces* sp.	JQ682622	0.22	3	3	+	−	0.66	−	−	−	+	−	R,S,C	10,12,17,21
5	CAI-78	*Streptomyces* sp.	JQ682623	0.95	0	2	+	−	2.92	+	−	+	+	−	R,S,C	10,12,17,21
6	CAI-85	*Streptomyces* sp.	KF770897	43.6	1	2	+	−	1.21	+	+	+	+	−	R,S,C	13,15,20,21
7	CAI-93	S. *fungicidicus*	KF742498	33.6	2	2	+	+	0	+	+	+	+	−	R,S,C	13,20,21
8	CAI-121	*Streptomyces* sp.	JN400113	43.7	3	2	+	+	0	+	−	−	+	−	R,S,C	9,14,16,19,21
9	CAI-127	*Streptomyces* sp.	JN400114	3.50	4	3	+	+	0	+	−	−	+	−	R,S,C	9,14,16,19,21
10	CAI-140	S. *coelicolor*	KF742497	15.4	1.3	3	+	−	0.353	+	+	+	+	−	R,S,C	13,20,21
11	CAI-155	*Streptomyces* sp.	KF770896	12.6	2	3	+	+	0.76	+	+	+	+	−	R,S,C	13,15,20,21,22
12	KAI-26	*Streptomyces* sp.	JQ682624	0.40	3	1	+	+	0.35	+	−	+	+	−	R,S,C	10,12,17,21
13	KAI-27	*Streptomyces* sp.	JQ682625	0.74	1	2	+	+	0.2	+	−	+	+	−	R,S,C	10,12,17,21
14	KAI-90	*Streptomyces* sp.	JN400116	0	3	3	+	+	0	+	−	+	+	−	R,S,C	9,14,16,19,21
15	KAI-180	*Streptomyces* sp.	KF742499	30.1	0	2	+	+	0	+	+	+	+	−	R,S,C	13,20,21
16	MMA-32	S. *roseoviolaceus*	JQ682626	4.66	3	2	+	+	0	+	+	+	+	+	R,S,C,P	10,11,18,21,23

Rating scale for siderophore are 0 = no change, 1 = positive, 2 = halo zone of 1 3 mm, 3 = halo zone of 4 6 mm and 4 = halo zone of 7 mm and above. ^#^Ratings scale for HCN production are 0 = no color change; 1 = light reddish brown; 2 = medium reddish brown and 3 = dark reddish brown.! The rating scale for Chitinase and Cellulase are 0 = no change; 1 = 1 6 mm; 2 = halo zone of 7 12 mm; 3 = halo zone of 19 24 mm and 4 = halo zone of 25 30 mm and above. Units One unit of β 1,3 glucanase activity is defined as the amount of enzyme that liberated 1 μmol of glucose h^−1^ at defined conditions. Foc *Fusarium oxysporum* f. sp. *ciceri*; Mp *Macrophomina phaseolina*; Rb – *Rhizoctonia bataticola*; Bc *Botrytis cinerea*; Sr *Sclerotium rolfsii*; R – Rice, S – Sorghum, C – Chickpea, P – Pigeonpea.

The biosynthetic potential of microorganisms genomes has been greatly underexplored^24^. For instance, many silent genes referred as cryptic or orphan are often present in the microbial genome pathways. Though, not all cryptic pathways are necessarily silent, some might have given lower rate of metabolite production under specified culturing conditions. This crucial reservoir can be untapped by whole genome sequence (WGS) data. For instance, *Streptomyces coelicolor* A3 (2) was known to produce four secondary metabolites until the WGS data have revealed the presence of additional 18 biosynthetic gene clusters^25^. The falling costs of WGS using next-generation sequencing (NGS) technologies has provided opportunity to catalogue genome wide variations present in any organism. Therefore, in the present study *de novo* genome assemblies of 16 *Streptomyces* strains have been developed using Illumina sequencing technology. These assemblies have been analyzed to identify the inter-species relationships, relevance of phenotypic and genomic data and additional insights of identified genome locus towards agriculturally important traits. Moreover, the correlation between the genetic makeup of these *Streptomyces* strains and their metabolites have provided the genes or biochemical pathways associated with phenotypic variability.

## Results

### *De novo* assemblies of sixteen *Streptomyces* strains

*De novo* assemblies were generated using shotgun sequencing. Each genome was sequenced from ~400 bp insert library to a coverage of ~500X using Illumina HiSeq. 2500. As a result, a total of 17 to 32 million paired-end reads were generated, yielding about 4.5 to 8 Gigabases (Gb) of sequence data per strain (Table 2). The sequencing reads were processed and assembled using *de novo* assembler SPAdes^26^, and the contigs with poor support from mapped reads were removed from analysis (Supplementary Fig. S1). As a result, total length of the final assemblies of 16 strains ranged from 6.8 to 8.31 Mb (Table 2), showing consistency with genome size estimates in *Streptomyces* strains. High congruency was found after mapping paired-end raw reads to the assembled contigs. These assemblies produced between 46 to 659 contigs, depending on the strain, with contig N50 ranging from 37 to 401 kb (Table 2). The average GC content of the *Streptomyces* strains was 72 to 73%.

**Table 2:** Details of genome sequencing and its assembly. The contigs (from de novo assembler SPAdes) having very low read support (<40) were dropped before generation of these statistics.

Strain no.	Paired-end reads (in millions)	Sequence data (in Gigabases)	Length of assembly (in Mega bases)	GC composition (in %)	No. of contigs	N50 (in Kb)	L50 (in Kb)
CAI-17	17.6	4.4	7.0	73	188	108	23
CAI-21	22.6	5.7	7.0	73	331	66	30
CAI-24	24.6	6.1	7.7	72	187	118	20
CAI-68	28.0	7.0	8.2	72	74	279	10
CAI-78	27.8	6.9	7.2	73	448	45	47
CAI-85	30.0	7.5	8.3	72	281	148	15
CAI-93	18.3	4.6	6.8	73	97	196	12
CAI-121	27.4	6.9	8.2	72	77	277	10
CAI-127	29.4	7.4	8.2	72	259	108	24
CAI-140	28.6	7.2	7.5	72	335	77	26
CAI-155	22.0	5.5	7.6	72	56	374	8
KAI-26	28.5	7.1	7.7	72	53	367	7
KAI-27	27.1	6.8	7.2	73	659	37	54
KAI-90	32.0	8.0	8.2	73	87	366	10
KAI-180	30.0	7.5	6.8	73	94	182	11
MMA-32	30.1	7.5	7.7	72	46	401	7

Quality assessment of each assembly was performed through, sequence accuracy, gene-space coverage and alignment to protein database. Conserved sets of genes^27^ were used to estimate gene space content in the 16 *de novo* assemblies. The results showed an average gene space completeness between 94 to 99% across the 16 *de novo* assemblies (Table 3). The fraction of entire proteome in *de novo* assemblies displaying full length alignment (i.e., query coverage of>95%) to the RefSeq proteomes of *Streptomyces* ranged from 76 to 90% (Table 3).

**Table 3:** Assessment of assembly quality.

Strain no.	Minimal bacterial gene set occurrence (in %)	Proteins showing BLAST hits with >95% query coverage to RefSeq *Streptomyces* proteome (in %)	Protein WITHOUT significant BLAST hits to RefSeq *Streptomyces* proteome (in %)
CAI-17	97	90	4
CAI-21	96	89	5
CAI-24	99	87	4
CAI-68	98	84	5
CAI-78	98	85	4
CAI-85	96	76	6
CAI-93	99	87	4
CAI-121	98	84	5
CAI-127	98	84	5
CAI-140	94	83	6
CAI-155	99	87	4
KAI-26	99	86	5
KAI-27	97	85	5
KAI-90	99	80	4
KAI-180	99	85	5
MMA-32	99	86	5

### Annotation and relationships in *Streptomyces* genomes

Genome assemblies contain 6,001 to 7,455 open reading frames (ORFs); we could assign a putative function through ‘Rapid Annotation using Sub-system Technology (RAST; http://rast.nmpdr.org/) to the encoded proteins for 67 to 73% of these (Table 4); the remaining were either hypothetical proteins or proteins of unknown or doubtful function. About 4–6% of the proteome of 16 strains failed to show any significant homology to the publicly available *Streptomyces* proteome sequences (Table 3). Curated annotation, involving hierarchical annotation of the genes/proteins, was available only for one-third (30–33%) of them (Table 4).

**Table 4:** Annotation of genome assemblies. The prediction of genes and their annotation was done using online RAST server.

Strain no.	Coding genes	Genes with annotation (in %)	Subsystems (Biol. Process) assigned	Role (Molecular Functions) assigned	Genes with Curated annotation (in %)
CAI-17	6132	72	421	1348	33
CAI-21	6146	71	409	1262	31
CAI-24	6615	69	430	1377	32
CAI-68	7326	67	440	1423	31
CAI-78	6053	72	423	1352	33
CAI-85	7455	69	430	1397	30
CAI-93	6044	72	413	1330	33
CAI-121	7327	67	442	1427	31
CAI-127	7299	67	434	1417	31
CAI-140	6837	69	412	1328	30
CAI-155	6581	69	432	1369	32
KAI-26	6744	68	439	1398	32
KAI-27	6001	72	417	1330	33
KAI-90	7175	73	443	1439	32
KAI-180	6050	71	426	1366	33
MMA-32	6738	68	439	1397	32

### Comparison of genomes based on entire gene set

**Figure 1:**
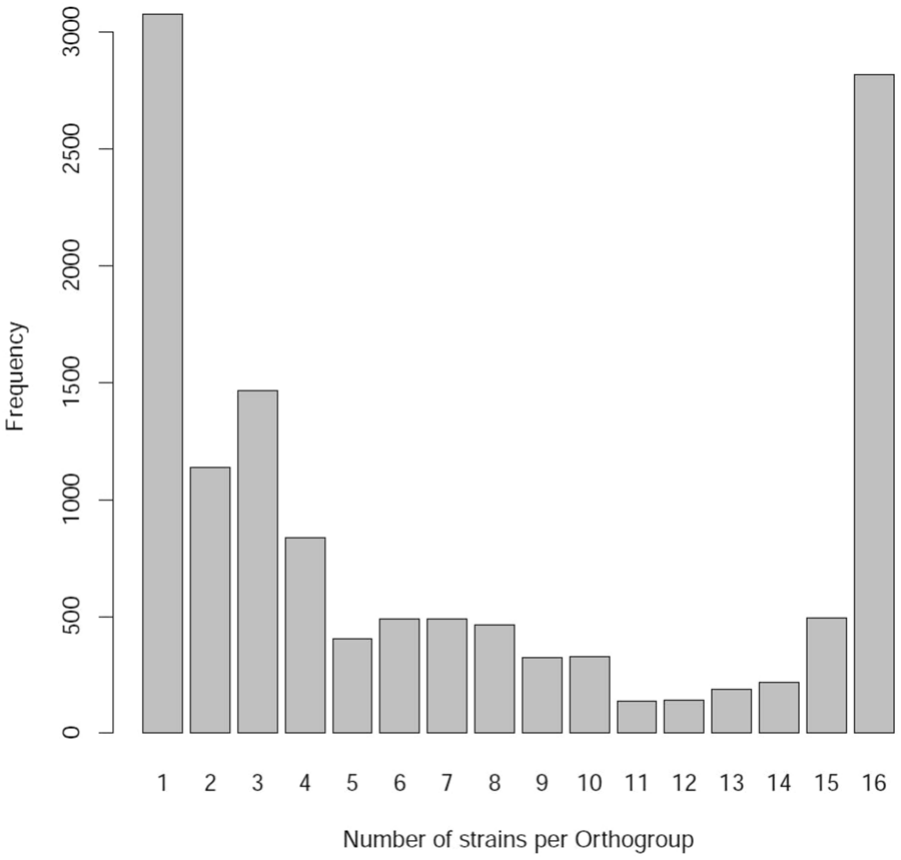
Frequency distribution of size of orthogroup (number of strains having one or more orthologs per orthogroup). The plot also displays genes which remained unassigned to any of the orthogroups (the leftmost bar).

**Figure 2:**
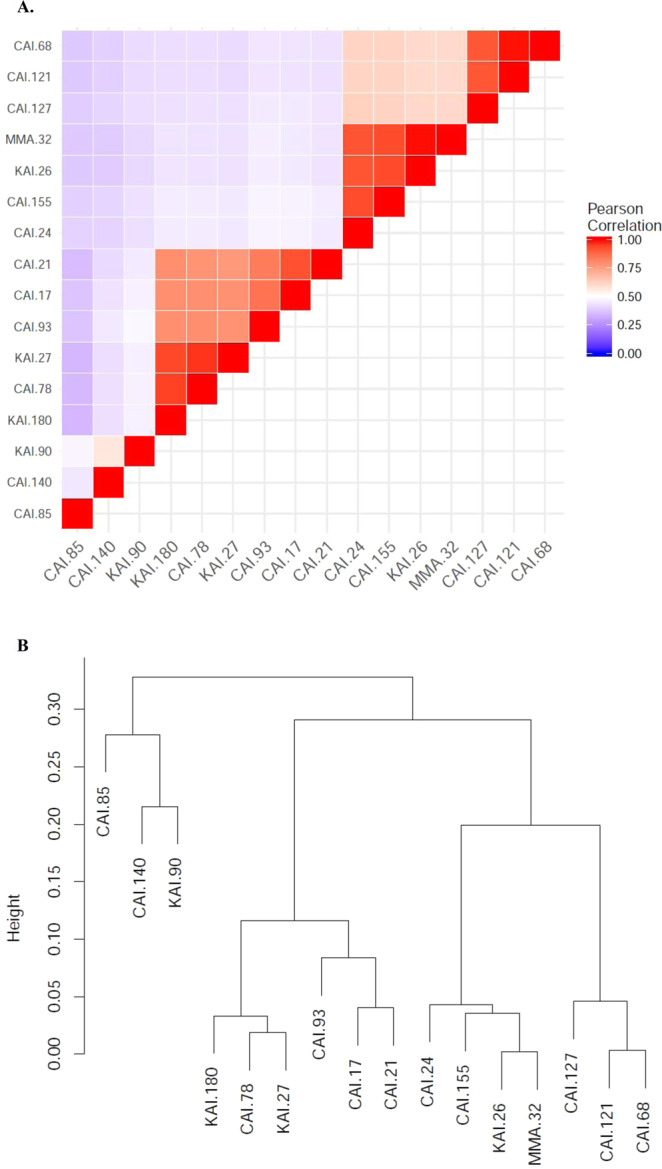
(**A**) Pearson correlation between all pairs of strains based on entire gene set including both ortholgous and unique genes. (**B**) Dendrogram based on same data. This pairwise gene-set comparison analysis indicated presence of 3 groups of closely related strains and one singleton.

Gene orthologs across 16 strains were identified using bi-directional best BLAST of peptide sequences along with phylogenetic analysis, as implemented in software package OrthoFinder^28^. While major fraction of the gene sets formed a total of 9,937 orthogroups, there remained about 3,078 genes from 16 strains unassigned to any of the orthogroups (Fig. 1). Among the orthogroups, over one fourth (28%) of them had member(s) present in all strains, indicating significant inter-genomic variation. Majority of the unassigned genes (~75%) were present in just three strains namely, CAI-85, CAI-140 and KAI-90, indicating the most diverged strains. The entire gene set of these strains were further compared pairwise for presence of common genes (i.e., orthologs present in the pair) and genes unique to each of them. A heatmap of correlation between all pairs of strains showed 3 distinct groups and one singleton (based on inter-nodal distance threshold of 0.2) (Fig. 2A,B). Two of three groups were relatively large (having 6 and 7 strains) compared to the third group having just 2 members (Fig. 2B).

The *de novo* assemblies of 16 strains were further compared with the publicly available *Streptomyces* species genomes/gene-sets available at NCBI/RAST database. These 16 strains were found closer to four public *Streptomyces* strains, namely, *S. griseus* NBRC 13350, *S. albus* J1074, *S. avermitilis* MA 4680 and *S. coelicolor* A3(2) (Table 5). Further, we quantified the extent of sequence similarity (in terms of fraction of orthologs) of one of the 16 strains with the closest reference *Streptomyces* species/strain. This pair-wise comparison have shown 70 to 85% of the genes were found common to the closest publicly available *Streptomyces* genomes (Table 5).

**Table 5:** Closest *Streptomyces* species/stains, and extent of overlap in terms of fraction of orthologs to total genes.

Strain no.	Closest public *Streptomyces* genome	Orthologs present in corresponding strains (%)
CAI-17	*Streptomyces albus* J1074	70
CAI-21	*Streptomyces albus* J1074	69
CAI-24	S. *griseus* NBRC 13350	80
CAI-68	S. *griseus* NBRC 13350	84
CAI-78	*Streptomyces albus* J1074	70
CAI-85	S. *avermitilis* MA-4680	NA
CAI-93	*Streptomyces albus* J1074	70
CAI-121	S. *griseus* NBRC 13350	84
CAI-127	S. *griseus* NBRC 13350	84
CAI-140	S. *coelicolor* A3(2)	75
CAI-155	S. *griseus* NBRC 13350	80
KAI-26	S. *griseus* NBRC 13350	80
KAI-27	*Streptomyces albus* J1074	69
KAI-90	S. *coelicolor* A3(2)	70
KAI-180	*Streptomyces albus* J1074	70
MMA-32	S. *griseus* NBRC 13350	80

### Comparison of genomes based on molecular function of the genes

To understand the large scale functional differences among 16 *Streptomyces* genomes, the assemblies were also compared using annotated/curated genes. Since multiple genes may perform same molecular-function/role, so comparison was done at the level of molecular-functions/roles across all strains (equivalently, such comparison will be at the level of orthogroup). As a result, we could identify ~1,850 molecular-functions/roles across these genomes (Supplementary Table S1). While ~50% of such molecular-functions/roles were conserved across the genomes of 16 *Streptomyces* strains, only ~10% were strain specific, and the rest molecular functions/roles were present in various combinations (Supplementary Fig. S2 & Supplementary Table S1).

Among the unique molecular functions/roles, >95% were limited to just three of the *Streptomyces* strains namely, CAI-85, CAI-140 and KAI-90 (Supplementary Table S2). These strain-specific molecular functions were involved in biological processes such as, iron acquisition and metabolism, siderophore biosynthesis, phosphate metabolism, auxin biosynthesis, antibiotic resistance and toxin biosynthesis (streptolysin). On the other hand, conserved roles/molecular functions of *Streptomyces* strains belonged to almost all biological processes listed (Supplementary Table S1).

The remaining molecular functions (~800), other than unique and conserved, were present in multiple *Streptomyces* strains, that is, in subsets of 16 strains (Supplementary Table S2). There were several instances when same subset of strains was positive for other molecular functions/roles belonging to a common subsystem or biological process. Such behavior was most likely due to arrangement of the genes in an operon. For example, five roles under the subsystem ‘histidine degradation’ were present in same 14 *Streptomyces* strains, and absent in two strains namely, KAI-27 and CAI-78. Further, we have also estimated the relatedness in *Streptomyces* strains genomes based on roles/molecular functions. Similar to the observations of comparison of complete gene sets, as mentioned above, these samples formed three groups and a singleton (Supplementary Fig. S3A,B). The smallest group and the singleton, comprised of three strains CAI-85, CAI-140 and KAI-90, was due to the occurrence of almost all unique functions within these three strains.

### Biosynthetic gene clusters (BGCs)

**Figure 3:**
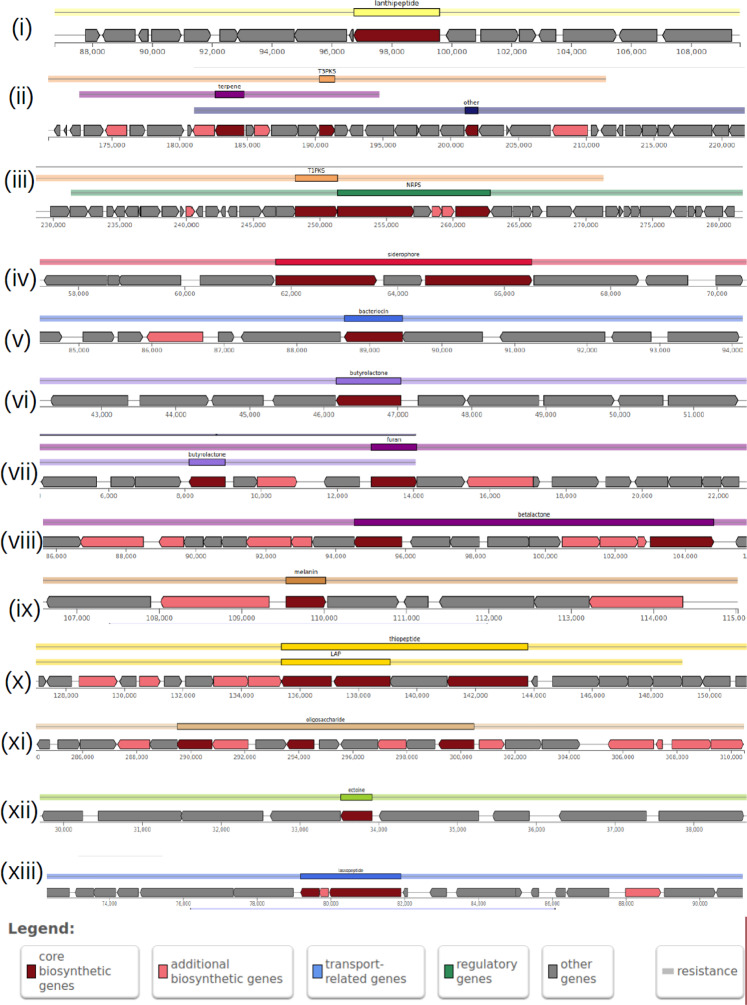
Graphical representation of key Biosynthetic Gene Clusters (BGCs) in one of the strains having maximum number of BGCs namely, CAI-121. Out of 39 BGCs present, only thirteen non-redundant ones are shown. The annotation of these BGCs are: (**i**) lantipeptide (**ii**) T3PKS, tarpene (**iii**) T1PKS, NRPS (**iv**) siderophore (**v**) bacteriocin (**vi**) butyrolactone (**vii**) furan (**viii**) betalactone (**ix**) melanin (**x**) thiopeptide, LAP (**xi**) oligosaccharide (**xii**) ectoine and (**xiii**) lassopeptide.

Since the *Streptomyces* genus is known for their ability to produce a variety of secondary metabolites, so the genome assemblies of these strains were predicted for presence of gene clusters involved in biosynthesis of secondary metabolites. While the number of BGCs per strain ranged from 23 to 39, the median number of genes per cluster ranged between 16 to 24, and the median size of BGCs ranged between ~22000 to ~33000 base pairs (Table 6; Fig. 3; Supplementary Table S3). On comparison to one of the closest publicly available genomes namely, *S. griseus* NBRC 13350, the above quantified properties appeared similar: presence of 40 BGCs, median number of genes per cluster was slightly higher (23), and median size of the BGCs was ~25000 base pairs (Table 6).

**Table 6:** Prediction of biosynthetic gene clusters (BGCs) in sixteen *Streptomyces* genome assemblies, and their comparison with the BGCs from one of the closest species, *S. griseus NBRC 13350*.

Strains	Number of biosynthetic clusters predicted	Distribution of number of genes per cluster (1st quartile, median, 3rd quartile)	Distribution of size of clusters (1st quartile, median, 3rd quartile; in base pairs)
CAI-21	24	13, 21, 28	15000, 28000, 42000
CAI-24	32	13, 20, 40	15000, 24000, 41000
CAI-68	39	13, 21, 35	19000, 26000, 47000
CAI-127	39	11, 21, 31	13000, 26000, 39000
CAI-140	23	11, 16, 25	12000, 22000, 31000
CAI-155	32	16, 23, 40	21000, 27000, 43000
KAI-90	35	10, 19, 32	13000, 22000, 40000
CAI-78	32	7, 18, 23	11000, 22000, 40000
CAI-85	31	11, 22, 38	12000, 23000, 49000
CAI-93	25	9, 20, 38	15000, 27000, 44000
CAI-121	39	13, 21, 35	19000, 26000, 47000
KAI-27	36	7, 17, 22	11000, 22000, 38000
KAI-180	26	6, 15, 27	12000, 23000, 32000
MMA-32	32	17, 24, 42	22000, 30000, 48000
KAI-26	32	18, 24, 42	22000, 30000, 49000
*S. griseus* NBRC 13350	40	10, 23, 36	17000, 25000, 52000

Apart from distribution of BGCs, the conservation of biosynthetic clusters (BC) was also examined in these strains. A genome pair comprising CAI-68 strain and *S. griseus* NBRC 13350 were selected due to highest percentage of shared genes (Table 5). Out of the 40 experimentally/predicted biosynthetic clusters of *S. griseus* NBRC 13350 (see methods), while ~40% were found largely intact in CAI-68 assembly, however, ~50% showed structural changes (largely deletions in CAI-68 assembly of size ~4,000 to 1000,000 bp) either at the end or within the BC (Supplementary Table S4). Three such cases of deletions within homologous BC were tested for being observed due to any assembly artifact, however, they all were found genuine (Supplementary Table S4).

### Plant growth-promotion (PGP) and biocontrol traits

*De novo* genome assemblies of *Streptomyces* strains were analyzed to identify genes involved in key pathways related to PGP and biocontrol activities. We have analyzed genome assemblies for traits like, biosynthesis and/or release of siderophores, auxin, hydrocyanic acid, chitinase and cellulase.

#### Siderophores

The phenotyping data have shown that 14 out of 16 *Streptomyces* strains had siderophore producing capacity in the specified rating scale of 1–4; the strains CAI-78 and KAI-180 did not produce siderophore (Table 1). Further, we have examined genomic data with respect to siderophore production. A total of 31 molecular functions were annotated to be involved in siderophore production (Supplementary Table S5), and majority (19 out of 31) of the molecular functions were conserved across strains (Supplementary Table S5). However, a set of four gene functions namely, *Isochorismate synthase*, *2,3-dihydro-2,3-dihydroxybenzoate dehydrogenase*, *2,3-dihydroxybenzoate-AMP ligase* and *Ferric enterobactin-binding periplasmic protein*, were found in high siderophore producing strains (Supplementary Table S6; Table 7). Seven high siderophore scorers (CAI-127, CAI-121, CAI-68, CAI-155, CAI-24, MMA-32 and KAI-26), with mean score of 3, all belonged to a single clade in the phylogenetic tree described earlier (Table 7; Fig. 2B). Out of four genes mentioned above, the three genes were co-located, and were likely to be part of an operon, while the fourth gene (*Ferric enterobactin-binding periplasmic protein*) was physically apart (Supplementary Table S4; Table 7). One of the strains (KAI-90) from a different clade, however, also showed relatively high siderophore score, but possessed only one of the four genes namely, *2,3-dihydroxybenzoate-AMP ligase*. On the other hand, six of the low siderophore scoring (KAI-180, KAI-27, CAI-78, CAI-21, CAI-17 and CAI-93), with mean score of 1, belonged to a distinct clade of the phylogenetic tree, and none of the four gene functions were present in them (Table 7; Fig. 2B). The siderophore scores of these two contrasting clades was significantly different (p-value = 0.00069 for one-sided t.test).

**Table 7:** Association of functional composition with the siderophore production. Among 12 molecular functions annotated in RAST for siderophore related, the strains having a set of four molecular functions were high siderophore producers (score 3–4 with an exception of CAI-155). The name of four genes are mentioned in the text. It was also observed that strains showing high siderophore scores belong to same clade in phylogenetic tree (in Figs. 2B and S3B).

Strain No.	Score for siderophore	Occurrence 4-siderophore related genes	Phylogenetic group the strains belong to
CAI-127	4	All 4	7 member clade
CAI-121	3	All 4	- do -
CAI-68	3	All 4	- do -
CAI-155	2	All 4	- do -
CAI-24	3	All 4	- do -
MMA-32	3	All 4	- do -
KAI-26	3	All 4	- do -
KAI-180	—	—	6 member clade
KAI-27	1	—	- do -
CAI-78	—	—	- do -
CAI-21	1	—	- do -
CAI-17	2	—	- do -
CAI-93	2	—	- do -
CAI-85	1	—	singleton
CAI-140	1	—	2 member clade
KAI-90	3	One of the 4 g	- do -

#### Auxin

The phenotyping data have shown that all but one strain (KAI-90) possess the ability of producing auxin (IAA) (Table 1). Four strains were highest producers (CAI-85, CAI-121, CAI-93 and KAI-180; in the range 30–43 µg ml^−1^), two strains were moderate producers (CAI-140 and CAI-155; in the range 12 and 15 µg ml^−1^) and the rest strains were least producers (CAI-17, CAI-21, CAI-24, CAI-68, CAI-78, CAI-127, KAI-26, KAI-27 and MMA-32; in the range 0.3–5.9 µg ml^−1^). In order to correlate the phenotyping data with the genomic composition of strains, we have selected eight genes representing three alternate pathways of auxin biosynthesis for analysis (Table 8). Only one gene, encoding enzyme *Indole 3-acetaldehyde dehydrogenase*, involved in two auxin biosynthetic pathways (Indole 3-pyruvate and Tryptamine) was present in all 16 strains (Table 8). Another gene namely, *Amine/Tyramine oxidase*, belonging to Tryptamine pathway was present in all but two strains (Table 8). The highest number (five) of genes involved in auxin biosynthesis was present in CAI-85, and the phenotype data suggested the same strain (CAI-85) to be one of the highest producer of IAA compared to other strains studied (Table 8). Alternatively, homology search of entire protein sequences of 16 strains for orthologs of IAA biosynthesis enzymes reported in literature didn’t add much to the results mentioned above (Supplementary Table S7). Therefore, it can be proposed that the combination of five genes is associated with Auxin biosynthesis at high level in CAI-85.

**Table 8:** Orthologs of enzymes involved in auxin (IAA) biosynthetic pathways based on RAST annotation. The data is based on mapping of orthologs to tryptophan metabolic pathway using RAST annotation.

Pathway	Reaction	Enzyme catalyzing the reaction	No of strains having ortholog	Remarks
	Tryptophan →Indole 3-pyruvate	EC 2.6.1.99, 2.6.1.27, 1.4.3.2		
Indole 3-pyruvate		(Aminotransferase)	0	
	Indole 3- pyruvate→	EC4.1.1.43, 4.1.1.74 (Indole		
	Indole 3- acetaldehyde	pyruvate decarboxylase)	0	
	Indole 3- acetaldehyde→	EC 1.2.1.3, 1.2.3.7 (Indole 3-		
	Indole acetate	acetaldehyde dehydrogenase)	12	
		EC 4.1.1.28 (Trip		
	Tryptophan →Tryptamine	decarboxylase)	1	Only in CAI-85
	Tryptamine→ Indole 3-	EC 1.4.3.22, 1.4.3.4		absent in CAI-140
Tryptamine	acetaldehyde	(Amine/Tyramine oxidase)	14	& KAI-90
	Indole 3-acetaldehyde→	EC 1.2.1.3, 1.2.3.7 (Indole 3-		
	Indole acetate	acetaldehyde dehydrogenase)	16	
	Tryptophan→ Indole 3-	EC 1.13.12.3 (Tryptophan		Present only in
Indole 3-acetamide	acetamide	mono-oxygenase)	1	KAI-90
	Indole 3- acetamide→			Absent in largest
	Indole acetate	EC 3.5.1.4 (IAM hydrolase)	9	clade

#### Hydro cyanic acid (HCN)

The phenotyping data generated on HCN production have been quantified on the scale of 1–3 (Table 1). All the strains were found to have HCN producing ability with KAI-26 as the least producer with a score of 1 (Table 1). Three genes (*hcnA*, *hcnB* and *hcnC*) corresponding to an operon have been reported in HCN biosynthesis in *Pseudomonas fluorescens* F113^29^. These three genes were used for bi-directional best BLAST to search their homologous sequences in 16 *Streptomyces* strains. We could detect homologous genes only for the *hcnC* in all the 16 *Streptomyces* strains (Supplementary Table S8). Nevertheless, a thorough examination of the BLAST results of *hcnA, hcnB* and *hcnC* genes indicated that a set of three co-localized genes appeared among top five BLAST hits in ten out of sixteen strains (Supplementary Table S9). The orthologs of *hcnA* was annotated as similar to *sarcosine oxidase* alpha subunit, *hcnB* as putative *oxidoreductase in 4 hydroxyproline* catabolic gene cluster, and *hcnC* as *D amino acid oxidase* (EC 1.4.3.3). Since these gene functions were not directly related to HCN biosynthesis, therefore, based on present results we could not establish a correlation between genotype and phenotyping data. These results indicate the *Streptomyces* strains use either a different biosynthetic pathway than the one present in *Pseudomonas*, or they use the above mentioned cluster of three co-localized genes.

#### Chitinase

As per the phenotypic results, chitinase production was observed in 11 strains such as, CAI-17, CAI-24, CAI-93, CAI-121, CAI-127, CAI-155, KAI-26, KAI-27, KAI-90, KAI-180 and MMA-32 (Table 1). The strains CAI-21, CAI-68, CAI-78, CAI-85 and CAI-140 were devoid the chitinase producing traits (Table 1). There were eleven gene functions mapped to Chitin and N-acetylglucosamine utilization subsystem. Nine out of eleven gene functions were present in all 16 strains (Supplementary Table S10). The two gene functions which were present in a subset of strains encoded for ‘Chitodextrinase precursor’ (EC 3.2.1.14) and ‘N-Acetyl- glucosamine ABC transport system, permease protein 2’.

#### Cellulase

Phenotyping for cellulase activity showed all strains were positive (Table 1). Cellulase activity involves two enzymes (Endoglucanase (EC 3.2.1.4)) and Beta-glucosidase (EC 3.2.1.21)). Therefore, we searched the genome sequence data for their homologous sequences in 16 studied strains. Interestingly we have identified both the enzymes homologues in all 16 strains (Table 9).

**Table 9:** Occurrence of enzymes involved in cellulase activity. The codes 0 and 1 indicates presence and absence of orthologs of the enzyme in a given strain. In three strains RAST couldn’t find an ortholog which was however predicted by Orthofinder (highlighted with blue).

Strain no	Endoglucanase (EC 3.2.1.4)	Beta-glucosidase (EC 3.2.1.21)	Phenotype
CAI-17	1	1	+
CAI-21	1	1	+
CAI-24	1	1	+
CAI-68	1	1	+
CAI-78	1	1	+
CAI-85	1	1	+
CAI-93	1	1	+
CAI-121	1	1	+
CAI-127	1	1	+
CAI-140	1	1	+
CAI-155	1	1	+
KAI-26	1	1	+
KAI-180	1	1	+
KAI-27	1	1	+
KAI-90	1	1	+
MMA-32	1	1	+

## Discussion

Relatively less number of actinobacterial genera relevant to agriculture have been studied at the whole genome level as compared to clinically-important genera e.g. *Mycobacterium*, *Propionibacterium*, etc. Hence, in the present study we have developed *de novo* assemblies for 16 *Streptomyces* strains, which were phenotypically characterized for their PGP, antagonistic and larvicidal (including one metabolite with insecticidal) activities against pathogens and insect pests of chickpea, pigeonpea and sorghum *in planta*^15,21,22^ (Table 1). *De novo* assemblies of selected 16 *Streptomyces* strains led to better understanding of the molecular mechanisms of their PGP/antagonistic/entomopathogenic functions and provided opportunities to discover more secondary metabolites.

The phylogenetic analysis conducted in the present study has demonstrated a much more accurate view of the species/strain phylogeny in *Streptomyces* that reflects different parts of the genome. Comparative analysis of gene annotations across the *Streptomyces* strains revealed many apparent lineage-specific gene families that might have emerged in the common ancestor of *Streptomyces* clade. The selected 16 *Streptomyces* strains have also shown to produce hydrolytic enzymes/harmones (as PGP and biocontrol traits) such as siderophore, indole acetic acid, hydrocyanic acid, chitinase, cellulase, protease, lipase and β-1,3-glucanase under *in vitro* conditions (Table 1). A number of genes/gene functions have been found associated with above mentioned traits in the present study. For instance, a set of four gene functions (*Isochorismate synthase*, *2,3-dihydro-2,3-dihydroxybenzoate dehydrogenase*, *2,3-dihydroxybenzoate-AMP ligase* and *Ferric enterobactin-binding periplasmic protein*) were found in high siderophore producing strains such as CAI-127, CAI-121, CAI-68, CAI-155, CAI-24, MMA-32 and KAI-26. Similarly^30^ reported five gene clusters for siderophore biosynthesis in *Streptomyces* sp.^31^ reported another siderophore gene cluster, 2,3-dihydroxybenzoate, for the first time in *Streptomyces* sp. ATCC 700974, which was also observed in this study. Siderophores are iron chelators secreted by bacteria, fungi and plants for their uptake, occur in several chemical forms: enterobactin (in *Escherichia coli*), aerobactin (in *Aerobacter aerogenes*), anguibactin, pyochelin and rhodotorulic acid (in *Rhodotorula pilimanae*) and ferrichrome (in *Ustilago maydis*)^32^. Siderophores also forms stable complexes with heavy metals such as U, Np, Al, Cu, Cd, Ga, Zn and Pb and increases the soluble metal concentrations^23^. This process helps to alleviate the heavy metal stresses in soils. Functional characterization (gene expression based) of this phenotype was earlier reported for all strains^13,15,17,19^. In brief, all the strains were grown separately in Bennett’s broth at 28 °C for 72 h in laboratory conditions and RNA was extracted to perform quantitative real time- PCR (qRT-PCR). Varying level of expression of siderophore synthetase (conserved across the strains) was observed, and the expression level correlated well with the siderophore production level, with exception in three cases^13,15,17,19^ (Supplementary Table S11). The expression results indicates that in addition to genetic makeup, this PGP trait may also be regulated at the transcriptional level.

Another PGP agent or plant growth hormone, i.e. Auxins, were also searched in the present data. Auxin producing bacteria are known to stimulate seed germination, root formation and root proliferation, thereby providing the host plant greater access to water and soil nutrients^33,34^. Several pathways exist in bacteria for auxin biosynthesis and in few of the cases tryptophan has been used as a precursor. In the present study, we have identified five genes associated with auxin biosynthesis and the pathway involving indole-3-pyruvate. Genes involved in another pathway for auxin biosynthesis i.e. pathway involving tryptamine were also searched in the present data. In tryptamine pathway three enzymes would be required and two of them are unique to it. Only one strain (CAI-85) in the present study had all the enzymes, whereas in at least 14 strains, enzymes catalyzing last two reactions were present. The third pathway involved in auxin biosynthesis i.e. indole-3- acetamide as a precursor and two enzymes were looked for their presence in the targeted strains. Sequence data showed that in just one strain (KAI-90) both the enzymes were present, and in about 9 strains only the enzyme catalyzing second reaction was present (Supplementary Table S7). Similar to present findings, *Streptomyces* sp. such as *S. violaceus*, *S. scabies*, *S. griseus*, *S. exfoliates*, *S. coelicolor* and *S. lividans* synthesize IAA in the presence of tryptophan *via* indole-3-acetamide pathway^35,36^. In addition to indoleacetamide hydrolase pathway nitrilase pathway has also reported in *Streptomyces* sp. (GKU 895)^37^. Like siderophore, function characterization of IAA pathway by qPCR was earlier reported for almost all strains, showing correlated expression with the IAA production level^13,15,17,19^. While minimal gene expression was reported among the low IAA producers, as high as 24 fold up-regulation was found among the high IAA producers, with an exception of strain CAI-68^13,15,17,19^ (Supplementary Table S12). This behavior is indicative of regulation (of IAA production pathway) at transcriptional level.

Similar to above mentioned PGP or biocontrol agents, in the present study, few genes or molecular functions involved in HCN, chitinase and cellulase were also discovered in *Streptomyces* strains following candidate based approach. However, in the present study we could not establish association of genes with HCN, chitinase and cellulase synthesis. A whole genome transcriptome analysis in future together with *de novo* assemblies generated in the present study may help in identification of the candidate genes responsible for HCN, chitinase and cellulase.

## Conclusion

The present study has developed complete genome assemblies for 16 *Streptomyces* strains and provided better understanding of genomic composition, genetic similarity and information on genes associated with favorable traits. However, identified favorable traits associated genes in the present study needs to be validated through construction of knock-outs or gene expression analysis in future studies. This can be considered as limitation of the present study and an opportunity for the upcoming studies. Moreover, we anticipate advancements made in the present study will provide opportunities for genome mining particularly of biosynthetic gene clusters from these and other micro-organisms, cloning of target genes, heterologous expression etc.

## Materials and Methods

### PGP strains

Sixteen strains of *Streptomyces* (CAI-17, CAI-21, CAI-24, CAI-68, CAI-78, CAI-85, CAI-93, CAI-121, CAI-127, CAI-140, CAI-155, KAI-26, KAI-27, KAI-90, KAI-180 and MMA-32) isolated previously from various herbal vermicompost and reported as potential for the PGP in chickpea, pigeonpea, rice and sorghum and biocontrol of important pathogens of chickpea and sorghum (Table 1)^9–23^ were further studied.

### Isolation of DNA

DNA was isolated as per the protocols of^38^. In brief, *Streptomyces* strains were inoculated in starch casein broth (SCB) and incubated for 5 days at 28 °C. At the end of incubation, the cultures were centrifuged at 8,000* g* for 10 min at 4 °C and the cells washed twice with STE buffer (0.3 M sucrose, 25 mM Tris/HCl and 25 mM Na_2_EDTA, pH 8.0). One g of the pellet was re suspended in 8.55 ml STE buffer and 950 µl lysozyme (20 mg/ml STE buffer) and incubated for 20–30 min at 30 °C. This was followed by addition of 500 µl of 10% SDS (w/v) and 50 µl of protease (20 mg/ml) and the mixture was held at 37 °C for 1 h. At the end of incubation, 1.8 ml 5 M NaCl was added with gentle mixing to avoid shearing the DNA and 1.5 ml 10% (w/v) CTAB in 0.7 M NaCl (CTAB/NaCl solution) and incubated for 20 min at 65 °C. After the addition of CTAB, all the steps were carried out at room temperature. The lysate was extracted twice with an equal volume of phenol/chloroform/isoamyl alcohol (25:24:1, by vol) and centrifuged at 12,000* g* for 10 min. The aqueous phase was finally extracted with chloroform/isoamyl alcohol (24:1, by vol) and transferred to a fresh tube. This was followed by addition of 600 µl of propan 2 ol and DNA spooled out after 10 min. Alternatively, it was recovered by centrifugation at 12,000* g* for 10 min. The pellet was washed twice with 70% (v/v) ethanol, vacuum dried and dissolved in 2 ml TE buffer (10 mM Tris/HCl and 1 mM EDTA, pH 8.0). RNaseA (50 mg/ml) was added with incubation at 37 °C for 2 h. The sample was again extracted with phenol as described above. DNA was re precipitated from the aqueous phase with addition of 100 µl of 3 M sodium acetate (pH 5.3) and 600 µl of propan 2 ol. The DNA pellet was washed with 70% (v/v) ethanol, dried and dissolved in TE buffer.

### Sequencing, assembly and annotation

The genomic DNA was sequenced to generate paired end reads using Hiseq. 2500 platform with a target of ~500X coverage. The reads were assembled using *de novo* assembler SPAdes version 3.10.1^26^. The contigs were filtered for minimum size (500 bp) and minimum read support (40). To evaluate the integrity of samples, 5–10 longest contigs were compared using BLAST (version 2.4.0+) against reference genomes of bacteria (source: ftp://ftp.ncbi.nlm.nih.gov/genomes/refseq/bacteria/), and consistency in term of target sequence was examined for the top hits (upto two). Hits to targets other than genus *Streptomyces*, would be indicative of issues with the assembly and/or contamination, but none was observed in the 16 assemblies.

To annotate the contigs, the sequence data were uploaded to Rapid Annotation using Subsystem Technology (RAST) online server (http://rast.nmpdr.org/rast.cgi)^39^. The RAST server predicted genes, translation of protein coding genes, and their annotation. Whether the proteome of each sample covers the minimal bacterial proteins^27^, the protein sequence of 339 such genes were first obtained from uniprot database (www.uniprot.org) such that the sequences of majority were form *Streptomyces* species present in uniprot database. Homology search of database sequences against proteome of each of the sample was performed using NCBI-BLASTP, and hits with Evalue <1E-05 were considered significant. 24 of the 339 query sequences didn’t get a hit in any of the 16 strains, and were dropped from the list of minimal bacterial gene set.

Besides, for examining quality of gene/protein sequences, a set comprising 32 RefSeq proteomes of publicly available *Streptomyces* genus (https://www.ncbi.nlm.nih.gov/assembly?LinkName=genome_assembly&from_uid=13511), was used as a database against each strain using NCBI-BLASTP. Sequences without a hit or hit with Evalue > =1E-03 were removed. Further, for a significant hit, if the alignment length covered 95% or more of length of query sequence, then it indicated assembly of full gene (i.e., full-length alignment).

### Comparison of genomes using gene sets

To compare the genomes at gene level, two approaches were implemented. In first approach, entire protein sequences of all strains were subject to prediction of orthogroups using default parameters of OrthoFinder tool^28^. In second approach, the genes which were successfully annotated using Subsystem technology were grouped based on molecular function (role). This was equivalent to orthogroups predicted by OrthoFinder. For each molecular function, if one or more genes were present identified in a strain, then assigned a binary code of 1, otherwise 0 (Supplementary Tables S1–S3). To get a summary, these binary codes were summed to report number of strains having a molecular function (role) per orthogroups.

### Prediction of biosynthetic gene clusters (BGCs) and their conservation

The BGCs were predicted using standalone version of antiSmash-v5.0^40^ with following parameters:*–minimal,–genefinding-tool none*. For finding conservation of BGCs, those identified by IMG database in *S. griseus* NBRC 13350 genome were obtained (https://img.jgi.doe.gov/)^41^, and nucleotide sequences of BGC regions were extracted from the complete genome downloaded from NCBI-RefSeq database (ID: GCF_000010605.1_ASM1060v1). Homology of these sequences were searched against CAI-68 genome assembly using BLASTN (pvalue cutoff: 1E-150), and the high scoring pairs were arranged in the increasing order of genomic position to figure out any structural variations (i.e., large insertions, deletions and translocations). Three BGC cases where deletions were observed in CAI-68 genome with respect to *S. griseus* NBRC 13350 genome, their sequences extracted and were BLASTed online against the NCBI complete genome database, followed by evaluation of the query matched with any of the database sequences without any break (that is, no fragmented match).

### Discovery of candidate genes for PGP/biocontrol properties

To discover the genes underlying various PGP or biocontrol traits, multiple approaches were implemented. If the pathway or process was characterized in curated annotation of RAST or its pathways, then the genomic information was directly compared to the phenotype. If incomplete/nil data was found from RAST annotation, then KEGG pathway database (http://www.genome.jp/kegg/kegg2.html) was examined followed by searching orthologs of the KEGG enzymes in the peptide data through bi-directional Best BLAST. In case even KEGG database didn’t have any information, the literature was searched for genetic/genomic studies about that process. Only literature on bacterial species, in particular, in actinomycetes group were preferred. These analysis were often complemented by exploiting the orthogroups predicted by OrthoFinder.

## Supplementary information

Supplimentary information file.

## Data Availability

The sequencing data generated in this study has been submitted at National Centre for Biotechnology Information (NCBI) under the Bioproject ID PRJNA510915.
