# Supplementary material for: Complete genome sequence of sixteen plant growth promoting Streptomyces strains
Source: Sci Rep. 2020 Jun 24;10:10294. doi: 10.1038/s41598-020-67153-9 (PMC7314817; doi:10.1038/s41598-020-67153-9)
Supplement: Supplementary file 1 — Supplimentary information file. [file 41598_2020_67153_MOESM1_ESM.pdf]

Supplementary Information

Title: Complete genome sequence of sixteen plant growth promoting *Streptomyces* strains

Authors: Gopalakrishnan Subramaniam[1,#,*], Vivek Thakur[1,2,#,*], Rachit K Saxena[1,#,], Srinivas Vadlamudi[1], Shilp Purohit[1], Vinay Kumar[1], Abhishek Rathore[1] , Annapurna Chitikineni[1] & Rajeev K Varshney[1,*]

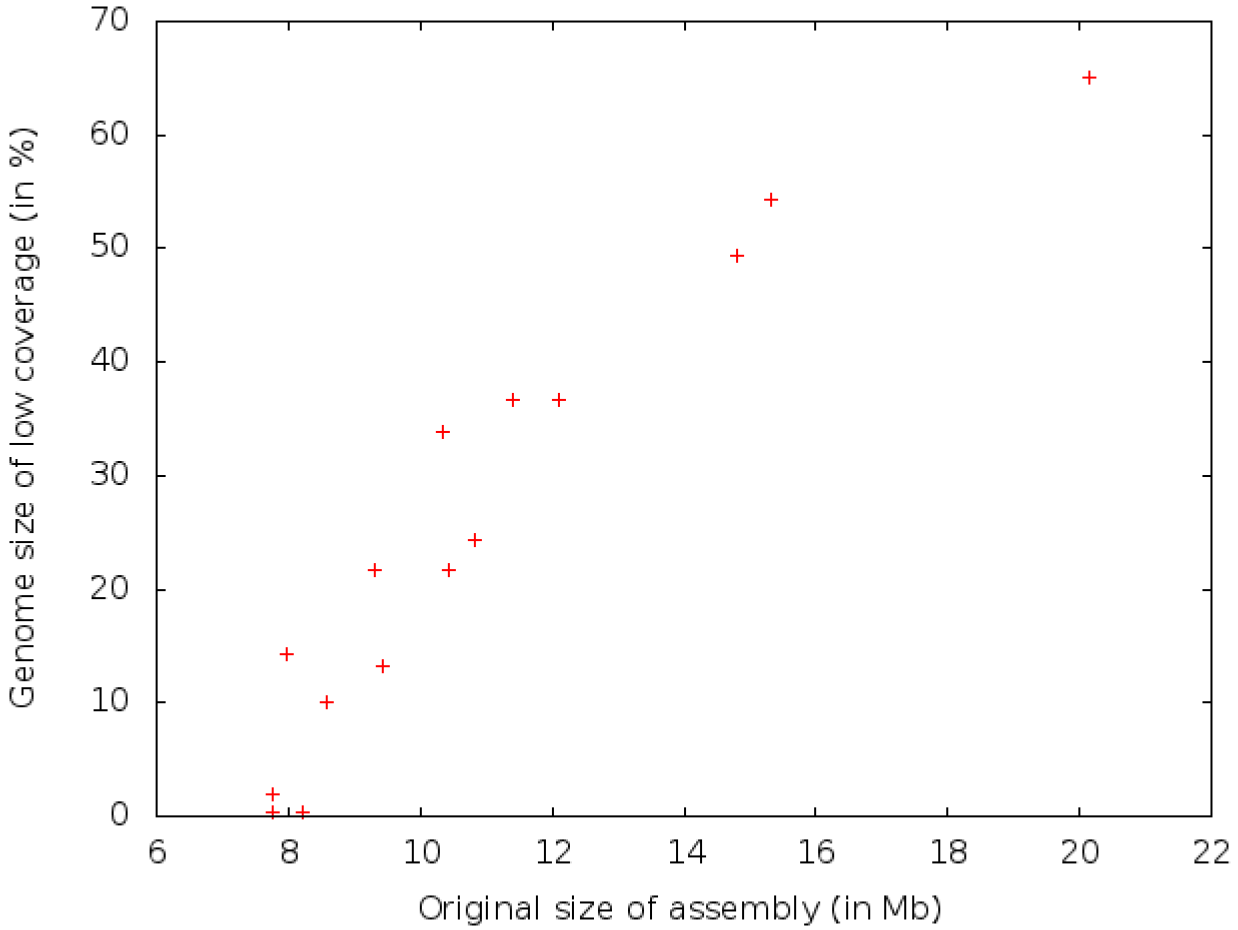

Figure S1. Size of raw assemblies increased linearly with increase in total length of low coverage contigs (support <40). The Y-axis shows the size of assembled genome comprised only of low coverage contigs, whereas the X-axis shows the total size of assembled genome.

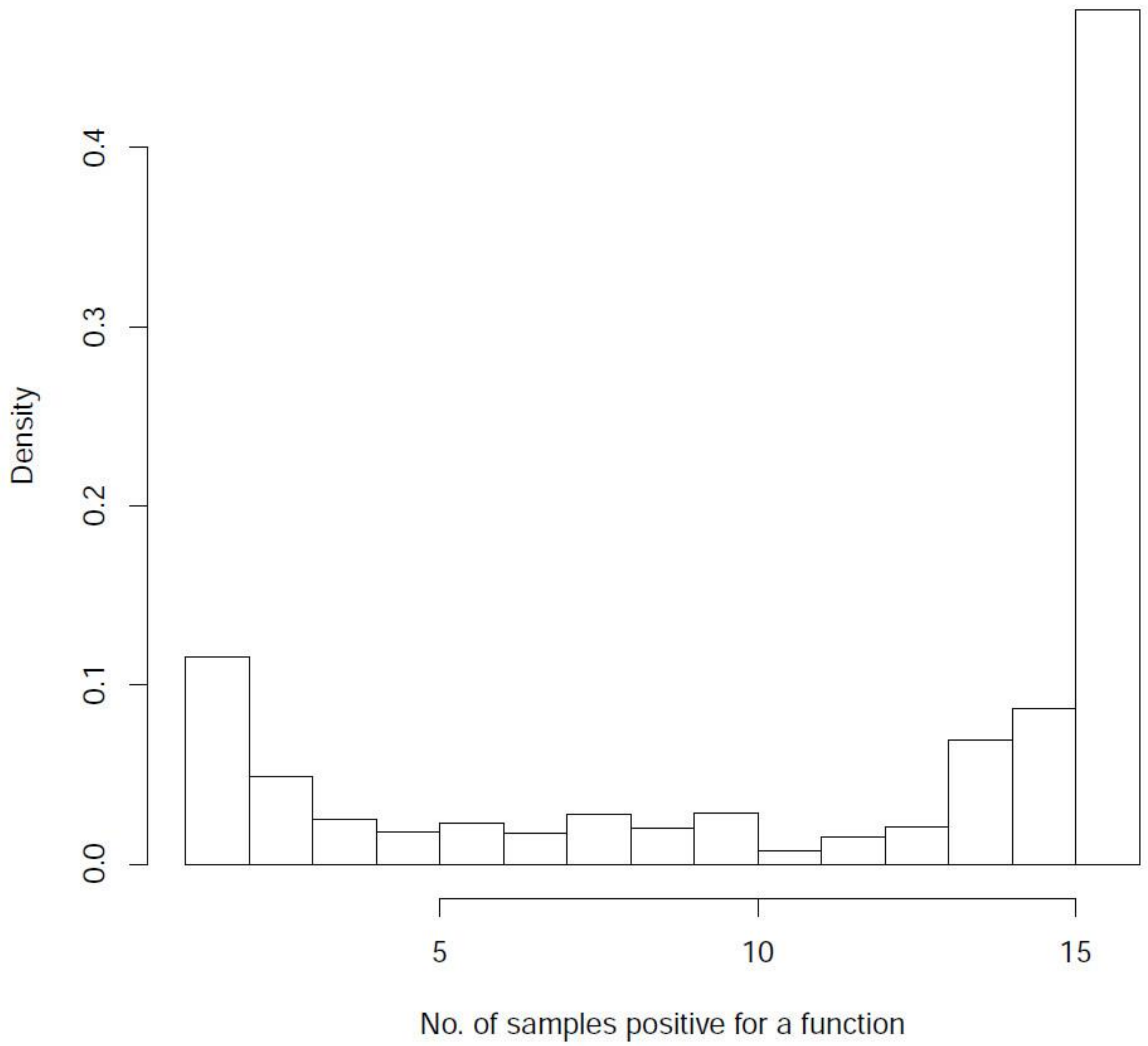

Figure S2. Frequency plot of number of strain(s) positive for a role/molecular function. The singletons are indicated by leftmost bar.

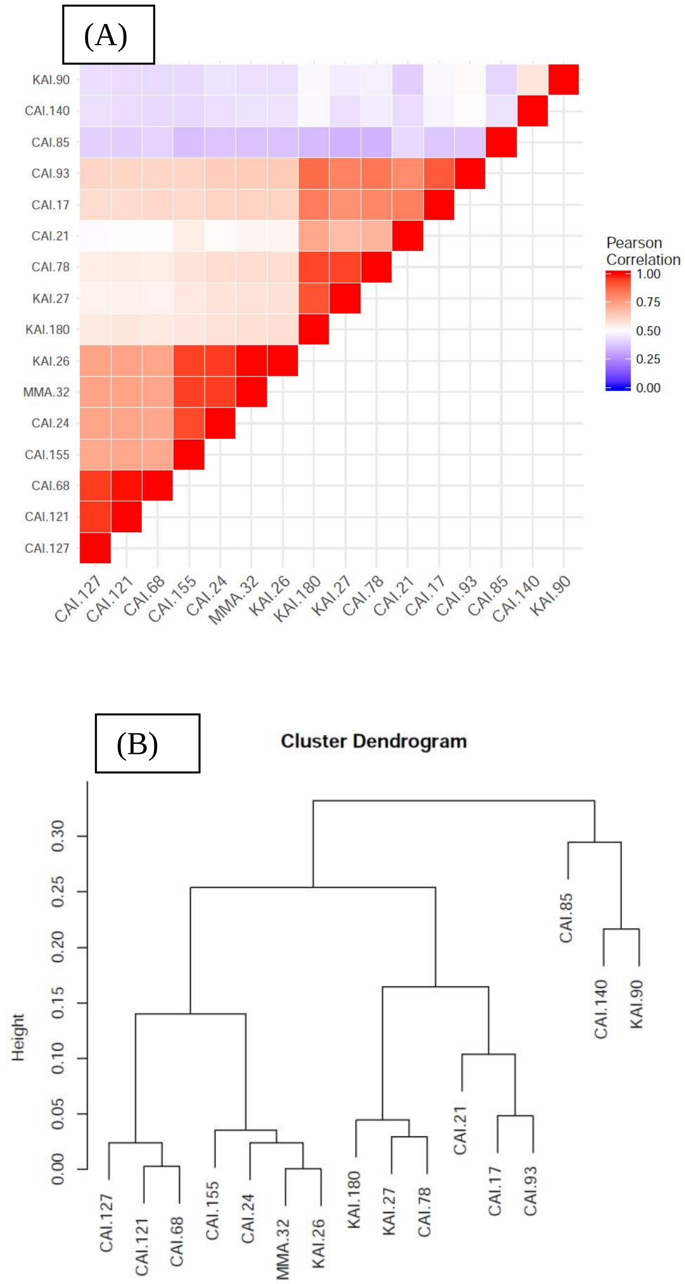

Figure S3. (A) Correlation among strains based on presence and absence of roles/molecular functions annotated by RAST. (B) A dendrogram based on same data.

Supplementary Table S1. Comparison of strains based on role/molecular-function assigned by RAST. First 4 columns (A to D) show annotation hierarchically, column E indicates number of strains per (ortho-) group, and columns F to U show strain wise data. The binary values (0/1) in columns F-U indicate absence and presence, respectively, of gene(s) encoding for the given role/molecular-function. Samples forming a group based on absence/presence of complete/partial subsystem are highlighted in yellow.
"Separate excel file has been uploaded."

Supplementary Table S2. Molecular functions unique to a strain. Column headers are same as in Supplementary Table S1. For easy identification, strain unique for a role/molecular function is highlighted in yellow.
"Separate excel file has been uploaded."

Supplementary Table S3. List of Biosynthetic clusters predicted in sixteen strains using antiSmash-v5.0 tool.
"Separate excel file has been uploaded."

Supplementary Table S4: Comparison of biosynthetic clusters between a reference genome and its closest strain, CAI-68. The known biosynthetic clusters are from S. griseus NBRC 13350 (genbank ID: NC_010572.1), and were obtained from IMG database, and were predicted by antiSMASH v5.0. Three of the internal deletions in BCG were BLASTed against microbial genomes to find if they are not assembly artifact.
"Separate excel file has been uploaded."

Supplementary Table S5. Occurrence of roles/molecular functions under siderophore category across sixteen strains. Column headers are same as in Table S1, except for splitting the strains in 4 groups based on dendrogram in Fig. 2 B or Fig. S3 B.
"Separate excel file has been uploaded."

Supplementary Table S6. Genomic position of 3 siderophore related genes present in 7 strains showed that they are colocalized, and they might be part of an operon.

		Genes and their location					
		2,3-dihydroxybenzoate-AMP ligase (EC 2.7.7.58)		Isochorismate synthase (EC 5.4.4.2)		2,3-dihydro-2,3-dihydroxybenzoate dehydrogenase (EC 1.3.1.28)	
**Strain**	**Contig ID**	**Start_pos (Kb)**	**End_pos (kb)**	**Start_pos (Kb)**	**End_pos (kb)**	**Start_pos (Kb)**	**End_pos (kb)**
CAI-127	NODE 3	29.5	31.2	31.2	32.4	32.5	33.3
CAI-121	NODE 9	20.9	21.1	20.8	20.9	20.7	20.8
CAI-68	NODE 9	20.9	21.1	20.8	20.9	20.7	20.8
CAI-155	NODE 2	20.7	20.9	20.6	20.7	20.5	20.6
CAI-24	NODE 2	15.3	15.5	15.2	15.3	15.1	15.2
MMA-32	NODE 4	32.7	32.9	32.9	33	33	33.1
KAI-26	NODE 3	32.7	32.9	32.9	33	33	33.1

Supplementary Table S7. Orthologs of enzymes involved in auxin (IAA) biosynthetic pathways based on orthology search. The data is based on bi-directional BLAST using probe sequences. The binary code 0 and 1 indicate absence and presence of enzyme.

“Separate excel file has been uploaded.”

Supplementary Table S8. Bi-directional BLAST search of hcn genes from *P. fluorescens* F113. All peptide sequences of *P. fluorescens* were searched against all peptides of a given strain, and vice versa. The hcnC gene had orthologs in all 16 strains. The hcnA and hcnB genes either failed to get a hit, or the best hit from the bi-directional BLAST conflicted each other. The IDs of peptide sequences of hcnA, hcnB and hcnC in RefSeq database were WP_014337863, WP_014337864 and WP_014337865, respectively.

Samples	Phenotypic score	Bi-directional best BLAST hit	Bi-directional best BLAST hit	Bi-directional best BLAST hit
**CAI-17**	3	NO HITS	NO HITS	1169742.4.peg.380
**CAI-21**	3	NO HITS	NO HITS	1169743.4.peg.53
**CAI-24**	3	NO HITS	Conflict	1076398.4.peg.4053
**CAI-68**	3	NO HITS	Conflict	1169745.4.peg.4333
**CAI-127**	3	NO HITS	Conflict	1076397.4.peg.1953
**CAI-140**	3	Conflict	Conflict	1902.20.peg.5440
**CAI-155**	3	NO HITS	Conflict	1472660.4.peg.2303
**KAI-90**	3	NO HITS	Conflict	1076400.4.peg.4966
**CAI-78**	2	NO HITS	NO HITS	1169746.4.peg.5436
**CAI-85**	2	Conflict	Conflict	1472662.4.peg.6082
**CAI-93**	2	NO HITS	NO HITS	68203.4.peg.2353
**CAI-121**	2	NO HITS	Conflict	1076396.4.peg.2432
**KAI-27**	2	NO HITS	NO HITS	1169748.4.peg.1766
**KAI-180**	2	NO HITS	NO HITS	53450.4.peg.743
**MMA-32**	2	NO HITS	Conflict	67360.4.peg.3453
**KAI-26**	1	NO HITS	Conflict	1169747.4.peg.3685

Supplementary Table S9. BLAST hits of hcnABC genes from P. fluorescens F113 to three colocalized genes. Ten out of sixteen strains showed presence of such sequentially located genes. Column 3 mentions the contig ID in which these sequential genes are present, columns 4-6 list the position of the genes in the given contig, columns 7-9 and 10-12 provides rank and Evalue required for assessing the quality of BLAST hits. In half cases having BLAST hits, the homology was restricted to just 2 genes. The rank and evalue indicated that these sequential genes could be biologically related to hcnABC genes.

			**hcnB**	**hcnA**	**hcnC**	**hcnB**	**hcnA**	**hcnC**	**hcnB**	**hcnA**	**hcnC**
**Strains**	**Phenotype score**	**Contig ID**	**Position of hits located sequentially (KB)**			**Rank of hits located sequentially**			**Evalue of hits located sequentially**		
**CAI-24**	3	NODE_20	8.2 – 10		6.7 – 7.8	1		2	4.80E-43		1.30E-31
**CAI-68**	3	NODE_7	93.2 – 94.8	92.9 – 93.2	91.8 – 92.9	1	1	2	7.50E-32	1.00E-08	4.30E-29
**CAI-127**	3	NODE_15	93.2 – 94.8	92.9 – 93.2	91.8 – 92.9	1	1	2	7.50E-32	1.00E-08	4.30E-29
**CAI-140**	3	NODE_17	71 – 72.4	72.4 – 72.6	72.6 – 73.8	1	1	2	1.90E-40	2.50E-11	1.20E-25
**CAI-155**	3	NODE_14	8 - 9.8		6.5 – 7.6	1		2	4.20E-44		4.00E-31
**KAI-90**	3	NODE_7	273 – 274.5	272.8 – 273.1	271.7 – 272.8	1	1	2	3.30E-23	2.00E-06	4.40E-27
**CAI-17**	3	NO HITS TO SEQUENTIAL GENES									
**CAI-21**	3	NO HITS TO SEQUENTIAL GENES									
**MMA-32**	2	NODE_17	8.1 – 9.8		6.5 – 7.7	1		2	1.60E-42		4.80E-32
**CAI-85**	2	NODE_7	92.2 – 93.7	93.7 – 93.9	93.9 – 95.1	1	1	2	6.50E-39	4.40E-13	1.40E-34
**CAI-121**	2	NODE_6	218.7 – 220.3	220.3 – 220.6	220.6 – 221.7	1	1	2	7.50E-32	1.00E-08	4.30E+29
**KAI-27**	2	NO HITS TO SEQUENTIAL GENES									
**KAI-180**	2	NO HITS TO SEQUENTIAL GENES									
**CAI-93**	2	NO HITS TO SEQUENTIAL GENES									
**CAI-78**	2	NO HITS TO SEQUENTIAL GENES									
**KAI-26**	1	NODE_17	8.1 – 9.8		6.5 – 7.6	1		2	1.60E-42		4.80E-32

Supplementary Table S10. Occurrence of gene functions mapped to “Chitin and N acetylglucosamine utilization” in 16 strains. The binary code 0 and 1 indicate absence and presence of gene function. The presence of a gene functions (code=1) across strains has been highlighted with light yellow background color.

			STRAINS															
			Clade with 7 members							Clade with six members						2 member clade		Singleton
**Subsystem**	**Molecular Function**	**Strains sharing gene function**	**CAI-127**	**CAI-121**	**CAI-68**	**CAI-155**	**CAI-24**	**MMA-32**	**KAI-26**	**KAI-180**	**KAI-27**	**CAI-78**	**CAI-21**	**CAI-17**	**CAI-93**	**CAI-140**	**KAI-90**	**CAI-85**
Chitin and N-acetylglucosamine utilization	Chitodextrinase precursor (EC 3.2.1.14)	8	1	1	1	1	1	1	1	**0**	**0**	**0**	**0**	**0**	**0**	**0**	1	**0**
Chitin and N-acetylglucosamine utilization	N-Acetyl-D-glucosamine ABC transport system, permease protein 2	12	1	1	1	**0**	**0**	**0**	**0**	1	1	1	1	1	1	1	1	1
Chitin and N-acetylglucosamine utilization	N-Acetyl-D-glucosamine ABC transport system, sugar-binding protein	15	1	1	1	1	1	1	1	1	1	1	1	1	1	**0**	1	1
Chitin and N-acetylglucosamine utilization	N-Acetyl-D-glucosamine ABC transport system, permease protein 1	16	1	1	1	1	1	1	1	1	1	1	1	1	1	1	1	1
Chitin and N-acetylglucosamine utilization	Beta-hexosaminidase (EC 3.2.1.52)	16	1	1	1	1	1	1	1	1	1	1	1	1	1	1	1	1
Chitin and N-acetylglucosamine utilization	Predicted transcriptional regulator of N-Acetylglucosamine utilization, GntR family	16	1	1	1	1	1	1	1	1	1	1	1	1	1	1	1	1
Chitin and N-acetylglucosamine utilization	N-acetylglucosamine kinase of eukaryotic type (EC 2.7.1.59)	16	1	1	1	1	1	1	1	1	1	1	1	1	1	1	1	1
Chitin and N-acetylglucosamine utilization	N-acetylglucosamine-6P-responsive transcriptional repressor NagC, ROK family	16	1	1	1	1	1	1	1	1	1	1	1	1	1	1	1	1
Chitin and N-acetylglucosamine	Chitin binding protein	16	1	1	1	1	1	1	1	1	1	1	1	1	1	1	1	1

utilization																		
Chitin and N-acetylglucosamine utilization	Predicted N-acetyl-glucosamine kinase 2, ROK family (EC 2.7.1.59)	16	1	1	1	1	1	1	1	1	1	1	1	1	1	1	1	1
Chitin and N-acetylglucosamine utilization	Chitinase (EC 3.2.1.14)	16	1	1	1	1	1	1	1	1	1	1	1	1	1	1	1	1
	**PHENOTYPE (Chitinolytic activity: present/absent)**		1	1	**0**	1	1	1	1	1	1	**0**	**0**	1	1	**0**	1	**0**

Supplementary Table S11: Functional validation of Siderophore production based on gene expression analysis. The table lists the strains for whom gene expression (using qPCR) was reported earlier. Varying level of expression of marker gene (Siderophore synthetase) was found in almost all strains (RNA polymerase principal sigma factor gene, HrdB, was used as control). With exception of 3 strains (highlighted in red), a very high correlation of >0.8 was observed between fold change in expression and siderophore production.

**Strain**	**Siderophore production (Ref: Table 1)**	**Fold change in gene expression**	**Reference**
CAI-127	4	12.4	19
CAI-121	3	12.6	19
MMA-32	3	12.2	15
CAI-68	3	10.8	17
CAI-24	3	6.2	19
CAI-93	2	1.4	13
CAI-17	2	0.1	17
CAI-85	1	4.5	13
KAI-27	1	3.6	17
CAI-21	1	1.98	15
CAI-140	1	0.1	13
CAI-78	0	1	17
KAI-26	3	1	17
KAI-90	3	0.1	19
CAI-155	2	25	13

Supplementary Table S12: Functional validation of IAA producing pathway/cluster based on gene expression analysis. The table lists 9 selected strains wherein gene expression analysis was reported in literature, and have contrasting IAA production levels. The expression was quantified using real time PCR for expression of Indole-3-acetaldehyde dehydrogenase gene, which catalyzes Tryptamine-mediated IAA biosynthesis.

Strain	IAA prod. (µg ml-1)	Fold change (qPCR based)	Reference
CAI-121	43.7	10x	19
CAI-85	43.6	24x	13
CAI-93	33.6	12x	13
CAI-78	0.95	Minimal expression	17
KAI-27	0.74	Minimal expression	17
KAI-26	0.4	2.5x	17
CAI-17	0.34	Minimal expression	17
CAI-68	0.22	13.7x	17
KAI-90	0	Minimal expression	19
